# Network meta-analysis on the effects of finerenone versus SGLT2 inhibitors and GLP-1 receptor agonists on cardiovascular and renal outcomes in patients with type 2 diabetes mellitus and chronic kidney disease

**DOI:** 10.1186/s12933-022-01676-5

**Published:** 2022-11-05

**Authors:** Yaofu Zhang, Li Jiang, Junheng Wang, Tongxin Wang, Chieh Chien, Weijun Huang, Xiaozhe Fu, Yonghua Xiao, Qiang Fu, Shidong Wang, Jinxi Zhao

**Affiliations:** 1 Dongzhimen Hospital, Beijing University of Chinese Medicine, Beijing, China; 2 Section II of Endocrinology & Nephropathy Department of Dongzhimen Hospital affiliated to Beijing University of Chinese Medicine, Beijing, China; 3 National Clinical Research Center for Chinese Medicine Cardiology, Xiyuan Hospital, China Academy of Chinese Medical Sciences, Beijing, China; 4 Department of Health Sciences and Medicine, University of Lucerne, Lucerne, Switzerland; 5 Key Laboratory of Chinese Internal Medicine of Ministry of Education and Beijing, Dongzhimen Hospital affiliated to Beijing University of Chinese Medicine, Beijing, China

**Keywords:** Finerenone, SGLT2 inhibitors, GLP-1 receptor agonist, Network meta-analysis, Cardiovascular outcome, Renal outcome, Type 2 diabetes mellitus, Chronic kidney disease

## Abstract

**Objective:**

To evaluate the cardiovascular and renal benefits of finerenone, sodium-glucose cotransporter-2 inhibitors (SGLT2i) and glucagonlike peptide-1 receptor agonists (GLP-1 RA) in patients with Type 2 Diabetes Mellitus (T2DM) and chronic kidney disease (CKD) with network meta-analysis.

**Methods:**

Systematic literature searches were conducted of PubMed, Cochrane Library, Web of Science, Medline and Embase covering January 1, 2000 to December 30, 2021. Randomized control trials (RCTs) comparing finerenone, SGLT-2i and GLP-1 RA in diabetics with CKD were selected. We performed a network meta-analysis to compare the two drugs and finerenone indirectly. Results were reported as risk ratio (RR) with corresponding 95% confidence interval (CI).

**Results:**

18 RCTs involving 51,496 patients were included. Finerenone reduced the risk of major adverse cardiovascular events (MACE), renal outcome and hospitalization for heart failure (HHF) (RR [95% CI]; 0.88 [0.80–0.97], 0.86 [0.79–0.93], 0.79 [0.67,0.92], respectively). SGLT-2i were associated with reduced risks of MACE (RR [95% CI]; 0.84 [0.78–0.90]), renal outcome (RR [95% CI]; 0.67 [0.60–0.74], HHF (RR [95% CI]; 0.60 [0.53–0.68]), all-cause death (ACD) (RR [95% CI]; 0.89 [0.81–0.91]) and cardiovascular death (CVD) (RR [95% CI]; 0.86 [0.77–0.96]) compared to placebo. GLP-1 RA were associated with a lower risk of MACE (RR [95% CI]; 0.86 [0.78–0.94]). SGLT2i had significant effect in comparison to finerenone (finerenone vs SGLT2i: RR [95% CI]; 1.29 [1.13–1.47], 1.31 [1.07–1.61], respectively) and GLP-1 RA (GLP-1 RA vs SGLT2i: RR [95% CI]; 1.36 [1.16–1.59], 1.49 [1.18–1.89], respectively) in renal outcome and HHF.

**Conclusions:**

In patients with T2DM and CKD, SGLT2i, GLP-1 RA and finerenone were comparable in MACE, ACD and CVD. SGLT2i significantly decreased the risk of renal events and HHF compared with finerenone and GLP-1 RA. Among GLP-1 RA, GLP-1 analogues showed significant effect in reducing cardiovascular events compared with exendin-4 analogues.

**Supplementary Information:**

The online version contains supplementary material available at 10.1186/s12933-022-01676-5.

## Background

As the prevalence of diabetes increases over the recent years, approximately 536.6 million are diagnosed with Dabetes Mellitus (DM). It is estimated that by the year of 2045, at least 783.2 million adults will be affected by diabetes [1]. Patients with diabetes are at high risk for adverse outcomes from atherosclerotic cardiovascular disease (ASCVD) [2, 3], heart failure and renal disease [4, 5]. With the increasing prevalence of Type 2 Diabetes Mellitus (T2DM) during recent decades, it has gradually become one of the primary factors accounting for the substantial global increase in end-stage renal disease (ESRD). Even with current therapies available [6–10], patients with T2DM and chronic kidney disease (CKD) still experience a significant cardiovascular and renal morbidity and mortality. Moreover, the risk of patients developing cardiovascular and renal events increase as DM and CKD progresses, potentially reaching renal and cardiac endpoint events such as ESRD, heart failure, myocardial infarction (MI) and stroke [11–14]. Therefore, the prevention of CKD progression and cardiovascular events is essential for the management of patients with T2DM and CKD.

Sodium-glucose cotransporter-2 inhibitors (SGLT2i) and glucagon-like peptide-1 receptor agonists (GLP-1 RA) were at the forefront of research in the field of diabetes. Several large cohort studies and randomized controlled trials (RCTs) have demonstrated cardiovascular and renal benefit for both drugs in patients with diabetes or kidney disease. Thus, the American Diabetes Association (ADA) recommended these two drugs for individuals with T2DM with or at high risk for ASCVD, heart failure, and/or CKD [15].

Finerenone is a nonsteroidal and selective mineralocorticoid receptor antagonist. According to two large randomized placebo-controlled trials targeted at T2DM and CKD patients, finerenone has been demonstrated to significantly reduce the occurrences of composite renal outcome (defined as a composite of a sustained decrease of at least 40% in the estimated glomerular filtration rate (eGFR) from the baseline, kidney failure, or death from renal causes) and composite cardiovascular outcome (defined as a composite of nonfatal MI, nonfatal stroke, death from cardiovascular causes, or hospitalization for heart failure [HHF]), regardless of patients with or without established cardiovascular disease [16, 17]. Consequently, in renin–angiotensin–aldosterone system (RAAS) inhibitions, finerenone represented a new frontier in the treatment of diabetic kidney disease [18]. ADA suggested that in patients with T2DM and CKD who were at increased risk for cardiovascular events or CKD progression or were unable to use the SGLT2i, finerenone was recommended to reduce CKD progression and cardiovascular events,. It was also suggested that the use of GLP-1 RA for individuals with T2DM with or at high risk of ASCVD, and/or CKD was optional [19].

Although finerenone, SGLT2i and GLP-1 RA offered cardiovascular or renal benefits to patients with T2DM and CKD, currently, there was no comparable study focusing on their effects on cardiovascular and renal outcomes. The network meta-analysis based on direct and indirect comparisons is an efficient algorithmically optimized method that can assist in clinical decision making. Even in the absence of head-to-head comparisons, it could still help to produce ranking results. Therefore, we herein investigate the effectiveness of finerenone, SGLT2i and GLP-1 RA in patients with T2DM and CKD by performing network meta-analysis based on RCTs.

## Methods

### Registration

We prospectively registered this systematic review in the International Prospective Register of Systematic Reviews database (PROSPERO) (registration number: CRD42022301457).

### Literature search

Our search strategy was conducted in accordance with the PRISMA (Preferred Reporting Items for Systematic Reviews and Meta-Analyses) extension statement for network meta-analysis [20, 21]. We performed a systematic search of PubMed, Cochrane Library, Web of Science, Medline and Embase from January 1, 2000 to December 30, 2021.

The following keywords were applied:((“Glucagon-Like Peptide 1 receptor[MeSH]” OR “GLP-1” OR “GLP1 receptor agonist” OR “glucagon-like peptide-1 receptor agonist” OR “Exenatide[MeSH]” OR “Liraglutide[MeSH]” OR “Lixisenatide” OR “Albiglutide” OR “Dulaglutide” OR “Semaglutide”) OR (“Sodium-Glucose Transporter 2 Inhibitors[MeSH]” OR “SGLT-2 inhibitor” OR “SGLT-2” OR “Canagliflozin[MeSH]” OR “Dapagliflozin” OR “Sotagliflozin” OR “empagliflozin” OR “Ertugliflozin” OR “Luseoglifozin”) OR “Finerenone”) AND ((“Renal Insufficiency, Chronic[MeSH]” OR “chronic kidney disease” OR “CKD” OR “kidney disease” OR “kidney failure” OR “chronic kidney failure” OR “renal failure” OR “chronic renal disease” OR “chronic renal failure” OR “CRF”) AND (“Diabetes Mellitus[MeSH]” OR “Diabetes Mellitus type 2” OR “type 2 Diabetes Mellitus”)).

The search results were screened separately by two blinded and independent authors (Z and J) to identify studies according to inclusion and exclusion criteria. When the two authors encountered the inconsistencies, a third author (W) was consulted to reach a decision. In addition, we reviewed the list of references included in the meta-analysis studies to minimize missing relevant studies.

### Study selection

Studies were selected if they met the following criteria: (1) they were published in peer-reviewed journals; (2) they included adult patients (≥ 18 years old) with T2DM and(or) CKD; (3) they were RCTs that compared finerenone, SGLT2i or GLP-1 RA with a placebo; (4) they compared the risk of cardiovascular and renal outcomes between treatment and placebo groups; and (5) they were published in English. Studies were excluded if data for estimating risk ratio (RR) was insufficient even after contact with the authors.

### Outcomes

Five outcomes were assessed in this study, which were MACE, Renal outcome, HHF, all-cause death (ACD) and CVD. The definition of MACE was a composite of CVD, nonfatal MI, or nonfatal stroke. If nonfatal MI and stroke data were unavailable, then the total MI and stroke were used instead. Renal outcome was defined as a composite of a sustained decrease of at least 40% in the eGFR from the baseline or a doubling of the serum creatinine level, kidney failure (a composite of end-stage kidney disease or sustained decrease in eGFR to < 15 ml/min/1.73 m^2^), or renal death. A similar renal outcome was used instead when this composite outcome was unavailable.

### Data extraction and quality assessment

Two researchers (Z and J), independently performed data abstraction and risk of bias assessment from eligible studies. Risk of bias assessment was performed according to the Cochrane risk of bias assessment tool (RoB 2.0) [22]. Any discrepancies in data extraction or quality assessment were resolved by a third reviewer (W). Data regarding cardiovascular and renal outcomes were abstracted from each study group. In this study, we also applied the Grading of Recommendations Assessment, Development, and Evaluation (GRADE) method in order to assess the quality of the evidence for each outcome, GRADE method can be found and accessed in GRADEpro GDT software [23]. Evidence quality was graded into four grades, these categories are labelled as High, Moderate, Low, and Very low. To prevent any other factors that may alter the result such as bias and inaccuracies, we have also referred to the five criteria, which are the risk of bias, the inconsistency, the indirectness, the imprecision and the publication bias. The application of these criteria is used as an evaluation to create the summary of evidence table [24, 25]. In addition to the five criteria, this network meta-analysis has also taken intransitivity and incoherence in to consideration, as they are vital when it comes to assess the quality of evidence for each outcome. In parallel, the quality of treatment effect estimation was rated based on the quality ratings of direct and indirect comparisons compliant to the GRADE Working Group approach [26].

### Statistical analysis

We performed a network meta-analysis using Stata (version 15.0). Risk ratio (RR) and 95% confidence interval (CI) were used to present the efficacy of treatments. The probability value of the I^2^ variable was calculated to assess heterogeneity, which was considered to be unimportant (0% < I^2^ < 40%), moderate heterogeneity (30% < I^2^ < 60%), substantial heterogeneity (50% < I^2^ < 90%), considerable heterogeneity (75% < I^2^ < 100%) [27].

In order to classified each of the intervention's effectiveness, finerenone, SGLT2i and GLP-1 RA were ranked from the most to the least effective or harmful, we used the Minimally Contextualized Framework to perform the results. The placebo was most closely connected to the other interventions and selected as the reference group, with an ineffective value, i.e. a relative effect value of 1, as the decision threshold. Based on the cardiovascular and renal outcomes, we used the 95% CI of the estimate of effect comparing each of the interventions against the placebo. If the interval crosses the decision threshold, then its corresponding intervention can remain in the same group as the placebo. On the other hand, if the interval did not cross the decision threshold, then depending on which side of the threshold the interval lies on, the intervention could be classified as more effective or less effective than the placebo. Based on comparisons made between pairs of interventions, should any intervention proves to be more effective than another category 1 intervention, then that corresponding intervention can be moved to a higher rated group (category 2) [28]. After evaluating the certainty of the evidence from finerenone and other 10 interventions included in SGLT2i and GLP-1 RA, the interventions were classified again into two broad categories: high certainty (moderate to high certainty evidence) and low certainty (low to very low certainty evidence). After checking consistency with pairwise comparisons and rankings, the intervention at the highest classification level could be considered as the most effective choice currently available, while low certainty as might be among the most effective.

We conducted a sensitivity analysis excluding “Cherney 2021”, as Cherney 2021 only included diabetics with severe CKD (eGFR: 15–30 ml/min/1.73 m^2^). In this network meta-analysis, none of the 5 outcomes had a closed loop. Therefore, it means that there was only indirect evidence among finerenone, SGLT2i and GLP-1 RA. Consequently, there was no need to test inconsistency for this network meta-analysis.

## Results

### Literature search and included studies

**Fig.1:**
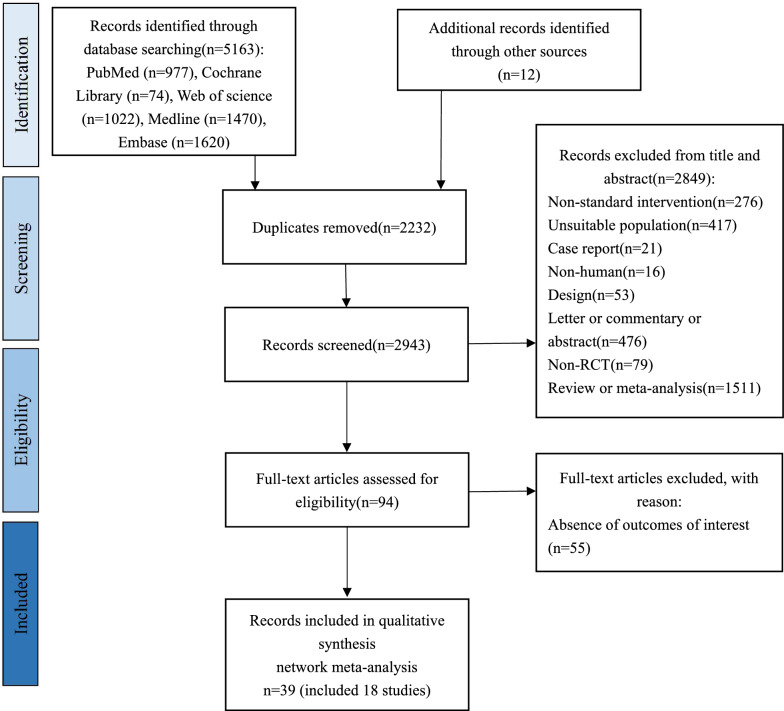
PRISMA flowchart

The detailed study filtering process is shown in Fig. 1. In brief, we retrieved a total of 5163 articles from PubMed (n = 977), Cochrane Library (n = 74), Web of science (n = 1022), Medline (n = 1470) and Embase (n = 1620) in primary search, during the process another 12 articles were identified through references. A total of 2232 duplicate articles were removed. After review by title and abstract, 2849 articles were removed due to: Non-standard intervention (n = 276), unsuitable population (n = 417), case report (n = 21), non-human (n = 16), design (n = 53), letter or commentary or abstract (n = 476), non-RCT (n = 79), review or meta-analysis (n = 1511). After that, 94 articles remained and entered into full-text assessing section. By assessing full text, 55 additional articles were excluded due to the lack of relevant outcome indicators. Finally, 39 articles (included 18 randomized controlled trials) were included in this network meta-analysis [7, 16, 17, 29–64]. Out of 18 studies, 3 studies were compared finerenone [16, 17, 29–31] with placebo; 8 studies were compared SGLT2i (Empaglifozin [32–36], Canaglifozin [7, 37–43], Dapaglifozin [44–48], Ertuglifozin [49–51], and Sotaglifozin [52, 53]) with placebo; 7 studies compared GLP-1 RA (Dulaglutide [54, 55], Albiglutide [56], Exenatide [57, 58], Semaglutide [59, 60], Liraglutide [61–63] and Efpeglenatide [64]) with placebo.

### Baseline characteristics of included studies in patients with T2DM and CKD

The characteristics of the included studies are presented in Table 1. The pooled population consisted of 51,496 patients with T2DM and CKD, 14,847 of them were in finerenone studies (7246 in the intervention group and 7601 in control group), 25,098 patients in SGLT-2i studies (13,260 in the intervention group and 11,838 in control group) and 11,551 patients in GLP-1 RA studies (5355 in the group treated with GLP-1 RA and 5796 in the control group). The definition of MACE in the included trials were consistent, except for four of them, EMPA-REG, DECLARE–TIMI 58, EXSCEL trials (data for nonfatal MI and stroke were not available, so we used total MI and stroke instead) and FIGARO-DKD (a composite of CVD, nonfatal MI, nonfatal stroke, or HHF). Whereas renal outcome were defined slightly different across included trials, but they were similar enough that can be used in analysis. The detailed definitions of renal outcome in different trials are shown in Table 2.

**Table 1:** Baseline characteristics of included studies in patients with T2DM and CKD

Trial	eGFR (ml/min/1.73 m^2^)	Number of patients			Age		Male		BMI		HbA1C (%)		eGFR (ml/min/1.73 m^2^)		Duration of diabetes	
		Total	I	C	I	C	I	C	I	C	I	C	I	C	I	C
*Finerenone vs placebo*																
FIDELIO-DKD	25 to < 75	6674	2833	3841	65.4 ± 8.9	65.7 ± 9.2	1953	2030	N/A		7.7 ± 1.3	7.7 ± 1.4	44.4 ± 12.5	44.3 ± 12.6	16.6 ± 8.8	16.6 ± 8.8
FIGARO-DKD	25 to 90	7352	3686	3666	64.1 ± 9.8		5107		31.4 ± 6.0		7.7 ± 1.4		67.8 ± 21.7		14.5 ± 8.5	
ARTS-DN	≥ 30	821	727	94	64.33 ± 9.20	63.26 ± 8.68	570	69	31.75 ± 5.57	32.49 ± 5.27	7.6 ± 1.3	7.6 ± 1.3	66.9 ± 21.9	72.2 ± 20.4	N/A	
*SGLT2i vs placebo*																
EMPA-REG	30 to 59	1819	1212	607	67.1 ± 7.6	67.1 ± 8.2	816	418	31.0 ± 5.5	30.9 ± 5.4	8.07 ± 0.86	8.03 ± 0.85	48.4 ± 8.2	48.6 ± 7.8	N/A	
CANVAS	30 to < 60	2039	1110	929	67.6 ± 7.8	67.6 ± 7.6	659	527	32.1 ± 5.9	32.5 ± 6.2	8.3 ± 1.0	8.3 ± 0.9	49.2 ± 7.8	49.0 ± 8.3	16.1 ± 8.4	15.7 ± 8.2
DECLARE-TIMI 58	< 60	1265	606	659	67.3 ± 6.6		814		34.5 ± 6.0			8.2 ± 1.2		51.4 ± 7.2	14.5 ± 8.9	
CREDENCE	30 to < 90	4401	2202	2199	62.9 ± 9.2	63.2 ± 9.2	1440	1467	31.4 ± 6.2	31.3 ± 6.2	8.3 ± 1.3	8.3 ± 1.3	56.3 ± 18.2	56.0 ± 18.3	15.5 ± 8.7	16.0 ± 8.6
VERTIS CV	30 to < 60	1807	1199	608	N/A		N/A		N/A			N/A		N/A	N/A	
DAPA-CKD	25 to 75	2906	1455	1451	64.1 ± 9.8	64.7 ± 9.5	961	980	30.2 ± 6.2	30.4 ± 6.3	7.8 ± 1.7	7.8 ± 1.6	44.0 ± 12.6	43.6 ± 12.6	13.7	13.8
SCORED	25 to 60	10,584	5292	5292	69	69	2945	2885	31.9	31.7	8.3	8.3	44.4	44.7	N/A	
Cherney 2021	15 to < 30	277	184	93	67.1 ± 9.8	68.0 ± 8.3	93	42	31.5 ± 5.8	31.7 ± 5.7	8.3 ± 0.9	8.4 ± 1.1	23.9 ± 4.6	24.1 ± 4.4	19.1 ± 9.2	20.7 ± 8.9
*GLP-1 RA vs placebo*																
LEADER	< 60	2158	1116	1042	67.3 ± 7.5	67.3 ± 7.5	691	631	32.6 ± 6.4	32.7 ± 6.6	8.7 ± 1.6	8.6 ± 1.5	45.5 ± 10.9	45.8 ± 10.8	15.4 ± 8.7	14.9 ± 8.5
REWIND	15 to < 60	2199	1081	1118	N/A		N/A		N/A		N/A		N/A		N/A	
HARMONY	30 to < 60	2222	1098	1124	N/A		N/A		N/A		N/A		N/A		N/A	
EXSCEL	30 to < 60	3177	1157	1620	66.5		1815		32.8		8.1		49.2		N/A	
PIONEER-6	30 to < 60	856	434	422	N/A		N/A		N/A		N/A		N/A		N/A	
SUSTAIN-6	< 60	939	469	470	N/A		N/A		N/A		N/A		N/A		N/A	
AMPLITUDE-O	< 71.5 mg/ml/1.73 m^2^	2218	1037	666	N/A		N/A		N/A		N/A		N/A		N/A	

*I* intervention, *C* control, *N/A* not available

**Table 2:** Definitions of terms in included studies

Trial	Study design	Patients enrolled in trials	Patients included in this study	Setting	Drug dose (mg/day)	Median follow up	eGFR	Range of HbA1c (%)	Definitions of renal outcome among included trials in patients with T2DM and CKD
*Finerenone vs placebo*									
FIDELIO-DKD	RCT	T2DM and CKD	T2DM and CKD	Multinational	Finerenone 10/20	2.6 years	25 to < 75	≤ 12	≥ 40% eGFR decline, renal death, ESRD, eGFR < 15 ml/min/1.73 m^2^
FIGARO-DKD	RCT	T2DM and CKD	T2DM and CKD	Multinational	Finerenone 10/20	3.4 years	25 to 90	≤ 12	≥ 40% eGFR decline, renal death
ARTS-DN	RCT	DN	DN	Multinational	Finerenone 1.25/2.5/5/7.5/10/15/20	90 days	≥ 30	≤ 12	≥ 40% eGFR decline
*SGLT2i vs placebo*									
EMPA-REG	RCT	T2DM	T2DM and CKD	Multinational	Empagliflozin 10/25	3.1 years	≥ 30	7 to 10	Macroalbuminuria, doubling of serum creatinine, eGFR < 45 ml/min/1.73 m^2^, renal-replacement therapy; renal death
CANVAS	RCT	T2DM	T2DM and CKD	Multinational	Canagliflozin 100/300	188.2 weeks	≥ 30	7 to 10.5	ESRD, renal death, ≥ 40% eGFR decline, doubling of serum creatinine
DECLARE–TIMI 58	RCT	T2DM	T2DM and CKD	Multinational	Dapagliflozin 10	4.2 years	CrCl ≥ 60 ml/min	6.5 to 12	≥ 40% eGFR decline, renal death, ESRD
CREDENCE	RCT	T2DM and CKD	T2DM and CKD	Multinational	Canagliflozin 100	2.62 years	30 to < 90	6.5 to 12	ESRD, doubling of serum creatinine level, renal death
VERTIS CV	RCT	T2DM	T2DM and CKD	Multinational	Ertugliflozin 5/15	3.5 years	≥ 30	7 to 10.5	N/A
DAPA-CKD	RCT	CKD	T2DM and CKD	Multinational	Dapagliflozin 10	2.4 years	25 to 75	N/A	≥ 50% eGFR decline, ESRD, renal-replacement therapy, eGFR < 15 ml/min/1.73 m^2^, renal death
SCORED	RCT	T2DM and CKD	T2DM and CKD	Multinational	Sotagliflozin 400	16 months	25 to 60	≥ 7	≥ 50% eGFR decline, renal-replacement therapy, eGFR < 15 ml/min/1.73 m^2^
Cherney 2021	RCT	T2DM and CKD	T2DM and CKD	Multinational	Sotagliflozin 200/400	52 weeks	15 to < 30	7 to 11	≥ 50% eGFR decline, renal-replacement therapy, eGFR < 15 ml/min/1.73 m^2^, renal death
*GLP-1 RA vs placebo*									
LEADER	RCT	T2DM	T2DM and CKD	Multinational	Liraglutide 1.8	3.84 years	N/A	≥ 7	Macroalbuminuria, doubling of serum creatinine, eGFR < 45 ml/min/1.73 m^2^, renal-replacement therapy, renal death
REWIND	RCT	T2DM	T2DM and CKD	Multinational	dulaglutide 1.5 weekly	5.4 years	≥ 15	≤ 9.5	Macroalbuminuria, ≥ 30% eGFR decline, renal-replacement therapy,
HARMONY	RCT	T2DM	T2DM and CKD	Multinational	Albiglutide 30/50	1.5 years	≥ 30	> 7	N/A
EXSCEL	RCT	T2DM	T2DM and CKD	Multinational	Exenatide 2 weekly	3.2 years	≥ 30	6.5 to 10	≥ 40% eGFR decline, renal-replacement therapy, renal death
PIONEER-6	RCT	T2DM	T2DM and CKD	Multinational	Semaglutide 14 oral	15.9 months	≥ 30	N/A	N/A
SUSTAIN-6	RCT	T2DM	T2DM and CKD	Multinational	Semaglutide 0.5/1 weekly	109 weeks	N/A	≥ 7	N/A
AMPLITUDE-O	RCT	T2DM	T2DM and CKD	Multinational	Efpeglenatide 4/6 weekly	1.81 years	N/A	> 7	Macroalbuminuria, ≥ 30% UACR increase, ≥ 40% eGFR decline, renal-replacement therapy, eGFR < 15 ml/min/1.73 m^2^,

*DN* diabetic nephropathy, *N/A* not available, *CrCl* creatinine clearance, eGFR (ml/min/1.73 m^2^), *UACR* urinary albumin-to-creatinine ratio

### Risk of bias

**Fig.2:**
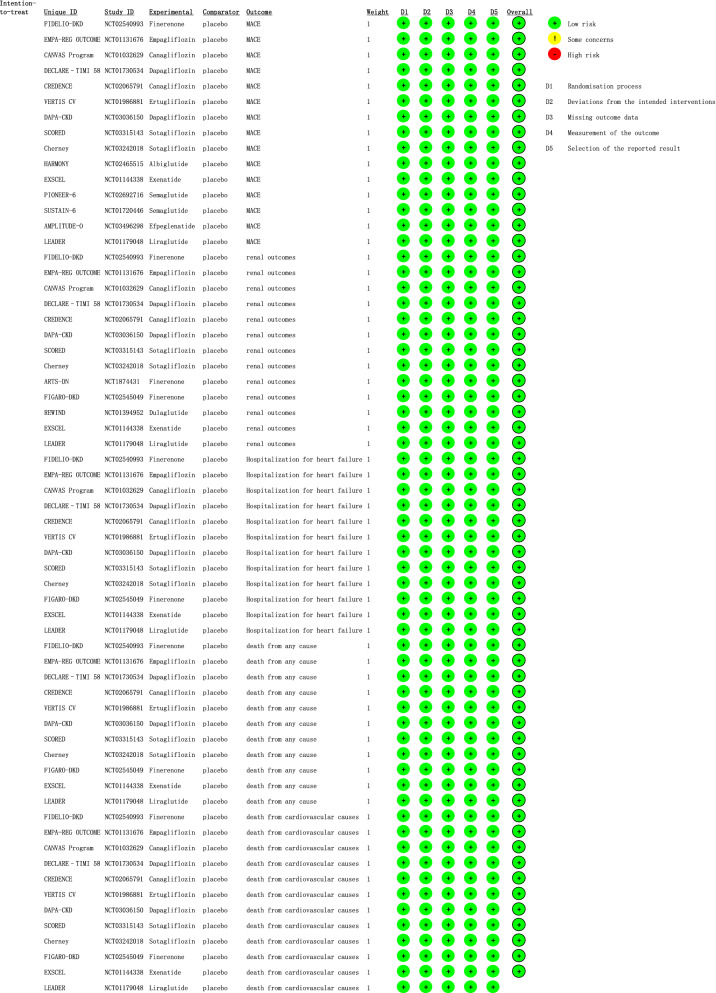
Detailed risk of bias in each study

We assessed the risk of bias in those trials using the Revised Cochrane Risk of Bias Tool (RoB 2.0). The quality evaluation of the included studies is shown in Fig. 2. All trials were evaluated as low risk in 5 outcomes. Detailed evaluations are as shown in Additional file 1 (RoB-2 evaluation) for each study.

### GRADE assessment

**Fig.3:**
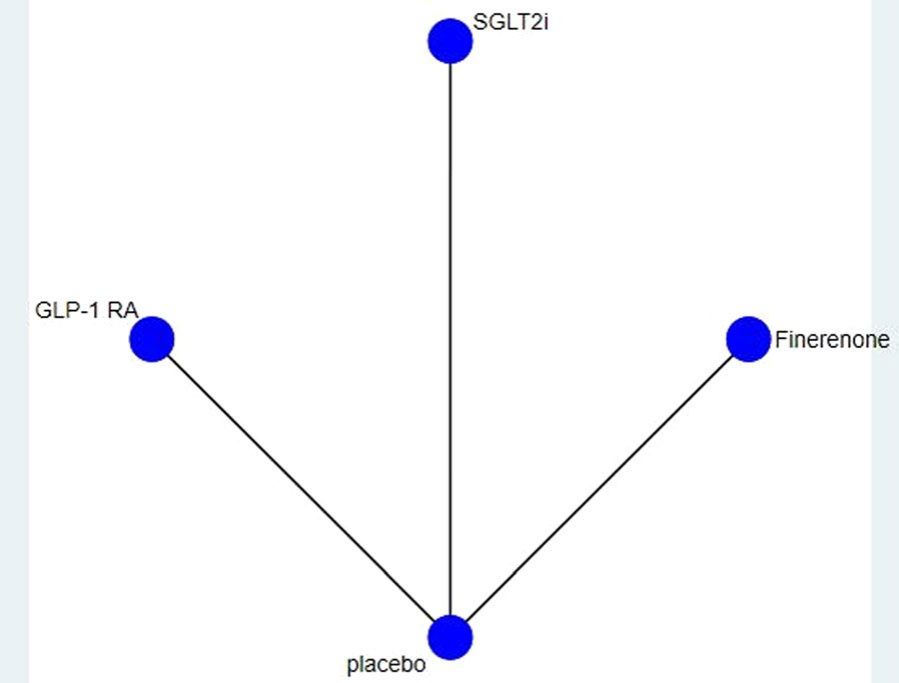
Network plot

In terms of reducing the MACE, there were 16 direct comparisons in the original articles and they were estimated high quality. In terms of renal outcome, there were 13 direct comparisons in the original articles whose estimated results were high quality. In terms of reducing the HHF and CVD, there were 12 direct comparisons in the original articles and they were rated as high quality. In terms of reducing the ACD, there were 11 direct comparisons in the original articles and they were rated as high quality. The detail was shown in Table 3. Figure 3 shows the network graph. As is shown in Additional file 2 (Publication bias), for the five outcomes, all studies were distributed symmetrically on both sides of the midline.

**Table 3:** GRADE assessment

Certainty assessment							No. of patients		Certainty	Importance
Intervention of studies	Study design	Risk of bias	Inconsistency	Indirectness	Imprecision	Publication bias	Intervention group	Control group		
MACE (No. of studies: 16)										CRITICAL
SGLT2i vs Placebo	Randomised trials	Not serious	Not serious	Not serious	Not serious	None	1255/13143 (9.5%)	1266/11688 (10.8%)	High	
GLP-1 RA vs Placebo	Randomised trials	Not serious	Not serious	Not serious	Not serious	None	740/6045 (12.2%)	795/5344 (14.9%)	High	
Finerenone vs Placebo	Randomised trials	Not serious	Not serious	Not serious	Not serious	None	686/6519 (10.5%)	777/6507 (11.9%)	High	
Renal outcome (No. of studies: 13)										CRITICAL
SGLT2i vs Placebo	Randomised trials	Not serious	Not serious	Not serious	Not serious	None	586/11731 (5.0%)	700/10981 (6.4%)	High	
GLP-1 RA vs Placebo	Randomised trials	Not serious	Not serious	Not serious	Not serious	None	428/3754 (11.4%)	474/3780 (12.5%)	High	
Finerenone vs Placebo	Randomised trials	Not serious	Not serious	Not serious	Not serious	None	861/7234 (11.9%)	997/6600 (15.1%)	High	
HHF (No. of studies: 12)										IMPORTANT
SGLT2i vs Placebo	Randomised trials	Not serious	Not serious	Not serious	Not serious	None	395/13144 (3.0%)	561/11689 (4.8%)	High	
GLP-1 RA vs Placebo	Randomised trials	Not serious	Not serious	Not serious	Not serious	None	174/2673 (6.5%)	191/2662 (7.2%)	High	
Finerenone vs Placebo	Randomised trials	Not serious	Not serious	Not serious	Not serious	None	256/6519 (3.9%)	325/6507 (5.0%)	High	
ACD (No. of studies: 11)										IMPORTANT
SGLT2i vs Placebo	Randomised trials	Not serious	Not serious	Not serious	Not serious	None	706/12128 (5.8%)	720/10898 (6.6%)	High	
GLP-1 RA vs Placebo	Randomised trials	Not serious	Not serious	Not serious	Not serious	None	339/2673 (12.7%)	378/2662 (14.2%)	High	
Finerenone vs Placebo	Randomised trials	Not serious	Not serious	Not serious	Not serious	None	552/6519 (8.5%)	614/6507 (9.4%)	High	
CVD (No. of studies: 12)										IMPORTANT
SGLT2i vs Placebo	Randomised trials	Not serious	Not serious	Not serious	Not serious	None	623/13144 (4.7%)	597/11689 (5.1%)	High	
GLP-1 RA vs Placebo	Randomised trials	Not serious	Not serious	Not serious	Not serious	None	213/2673 (8.0%)	235/2662 (8.8%)	High	
Finerenone vs Placebo	Randomised trials	Not serious	Not serious	Not serious	Not serious	None	322/6519 (4.9%)	364/6507 (6.0%)	High

According to recommendation of GRADE working group, we presented a four-step approach to rate the quality of evidence in each of the direct, indirect, and network meta-analysis estimates based on methods developed by the GRADE working group [26]. In this network meta-analysis, none of the 5 outcomes had a closed loop. Meaning that that no outcomes from both direct and indirect comparisons are included, rendering incoherence assessment unnecessary. The definition of renal outcome varied between studies included in this research, and the baseline eGFR of patients in the “cherney 2021” was different from other studies. For direct comparisons, “cherney 2021” included only 1% of patients in SGLT2i (277/25098). Therefore, risk of bias was not taken in to consideration. As for intransitivity, there was only indirect evidence in the intercomparison of finerenone, SGLT2i and GLP-1 RA. The GRADE working group recommends that situation regarding intransitivity may warrant particular attention, and the threshold for rating down for intransitivity may be lower [26]. Therefore, we downgraded the quality of evidence for the comparison between SGLT2i and finerenone, SGLT2i and GLP-1 RA. The detail was shown in Table 4.

**Table 4:** Estimates of effects and quality ratings for comparison of drugs to prevent cardiorenal outcomes

Comparison	Direct evidence		Indirect evidence		Network meta-analysis	
	RR [95% CI]	Quality of evidence	RR [95% CI]	Quality of evidence	RR [95% CI]	Quality of evidence
*MACE*						
SGLT2i vs Placebo	0.83 (0.77,0.90)	High	Not estimable^a^	–	0.84 (0.78,0.90)	High
GLP-1 RA vs Placebo	0.86 (0.78,0.94)	High	Not estimable^a^	–	0.86 (0.78,0.94)	High
Finerenone vs Placebo	0.89 (0.75,1.05)	High	Not estimable^a^	–	0.88 (0.80,0.97)	High
GLP-1 RA vs SGLT2i	–	–	1.03 (0.91,1.16)	Moderate^b^	1.03 (0.91,1.16)	Moderate^b^
Finerenone vs SGLT2i	–	–	1.06 (0.88,1.28)	Moderate^b^	1.05 (0.93,1.19)	Moderate^b^
Finerenone vs GLP-1 RA	–	–	1.03 (0.90,1.17)	High	1.03 (0.90,1.17)	High
*Renal outcome*						
SGLT2i vs Placebo	0.67 (0.60,0.74)	High	Not estimable^a^	–	0.67 (0.60,0.74)	High
GLP-1 RA vs Placebo	0.90 (0.80,1.02)	High	Not estimable^a^	–	0.90 (0.80,1.02)	High
Finerenone vs Placebo	0.86 (0.79,0.93)	High	Not estimable^a^	–	0.86 (0.79,0.93)	High
GLP-1 RA vs SGLT2i	–	–	1.36 (1.16,1.59)	Moderate^b^	1.36 (1.16,1.59)	Moderate^b^
Finerenone vs SGLT2i	–	–	1.29 (1.13,1.47)	Moderate^b^	1.29 (1.13,1.47)	Moderate^b^
Finerenone vs GLP-1 RA	–	–	0.95 (0.82,1.10)	High	0.95 (0.82,1.10)	High
*HHF*						
SGLT2i vs Placebo	0.60 (0.53,0.68)	High	Not estimable^a^	–	0.60 (0.53,0.68)	High
GLP-1 RA vs Placebo	0.90 (0.74,1.09)	High	Not estimable^a^	–	0.90 (0.73,1.09)	High
Finerenone vs Placebo	0.79 (0.67,0.92)	High	Not estimable^a^	–	0.79 (0.67,0.92)	High
GLP-1 RA vs SGLT2i	–	–	1.49 (1.18,1.89)	Moderate^b^	1.49 (1.18,1.89)	Moderate^b^
Finerenone vs SGLT2i	–	–	1.31 (1.07,1.61)	Moderate^b^	1.31 (1.07,1.61)	Moderate^b^
Finerenone vs GLP-1 RA	–	–	0.88 (0.68,1.14)	High	0.88 (0.68,1.14)	High
*CVD*						
SGLT2i vs Placebo	0.86 (0.77,0.96)	High	Not estimable^a^	–	0.86 (0.77,0.96)	High
GLP-1 RA vs Placebo	0.90 (0.75,1.08)	High	Not estimable^a^	–	0.90 (0.75,1.08)	High
Finerenone vs Placebo	0.88 (0.76,1.02)	High	Not estimable^a^	–	0.88 (0.76,1.02)	High
GLP-1 RA vs SGLT2i	–	–	1.04 (0.85,1.29)	Moderate^b^	1.04 (0.85,1.29)	Moderate^b^
Finerenone vs SGLT2i	–	–	1.02 (0.85,1.23)	Moderate^b^	1.02 (0.85,1.23)	Moderate^b^
Finerenone vs GLP-1 RA	–	–	0.98 (0.78,1.23)	High	0.98 (0.78,1.23)	High
*ACD*						
SGLT2i vs Placebo	0.90 (0.81,0.99)	High	Not estimable^a^	–	0.89 (0.81,0.99)	High
GLP-1 RA vs Placebo	0.89 (0.78,1.02)	High	Not estimable^a^	–	0.89 (0.77,1.02)	High
Finerenone vs Placebo	0.90 (0.80,1.00)	High	Not estimable^a^	–	0.90 (0.80,1.00)	High
GLP-1 RA vs SGLT2i	–	–	0.99 (0.84,1.18)	Moderate^b^	0.99 (0.84,1.18)	Moderate^b^
Finerenone vs SGLT2i	–	–	1.00 (0.86,1.16)	Moderate^b^	1.00 (0.86,1.16)	Moderate^b^
Finerenone vs GLP-1 RA	–	–	1.01 (0.85,1.20)	High	1.01 (0.85,1.20)	High

^a^Cannot be estimated because the drug was not connected in a loop in the evidence network

^b^Intransitivity

### Network meta‑analysis of treatment groups

#### MACE

**Fig.4:**
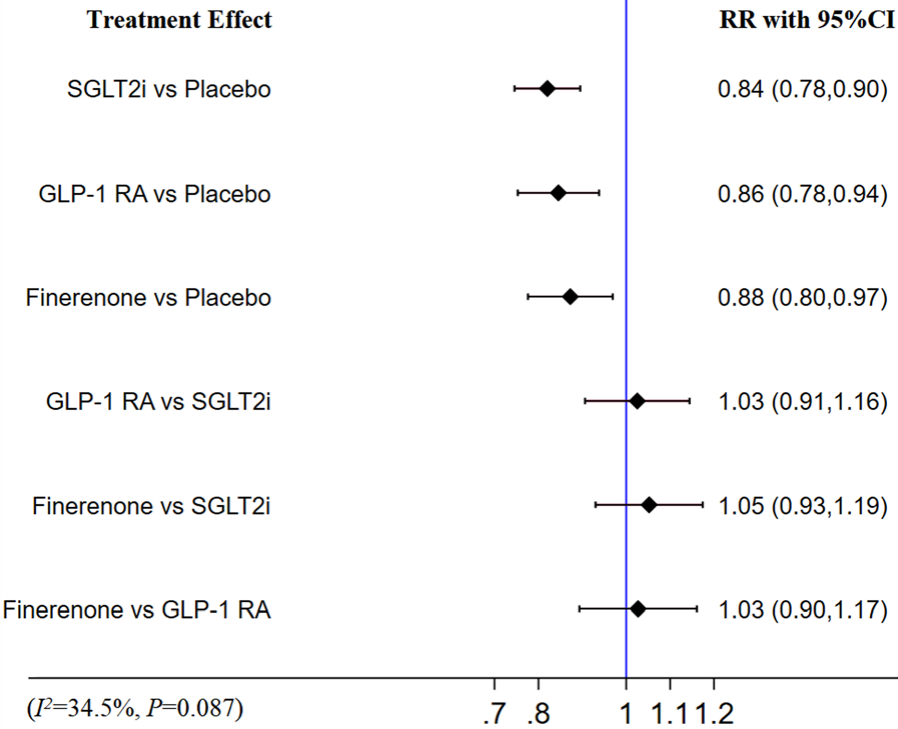
Network meta-analysis reporting RR for MACE in patients with T2DM and CKD

Compared with placebo, finerenone (RR [95% CI]; 0.88 [0.80–0.97]), SGLT-2i (RR [95% CI]; 0.84 [0.78–0.90]) and GLP-1 RA (RR [95% CI]; 0.86 [0.78–0.94]) were associated with a decreased risk of MACE. Finerenone didn`t show a significant difference in reducing the risk of MACE compared with SGLT-2i (RR [95% CI]; 1.05 [0.93–1.19]) and GLP-1 RA (RR [95% CI]; 1.03 [0.90–1.17]). There was also no significant difference in the risk of MACE between SGLT-2i and GLP-1 RA (RR [95% CI]; 1.03 [0.91–1.16]). There was no heterogeneity (I^2^ = 34.5%, p = 0.087). The detail is shown in Fig. 4.

### Renal outcome

**Fig.5:**
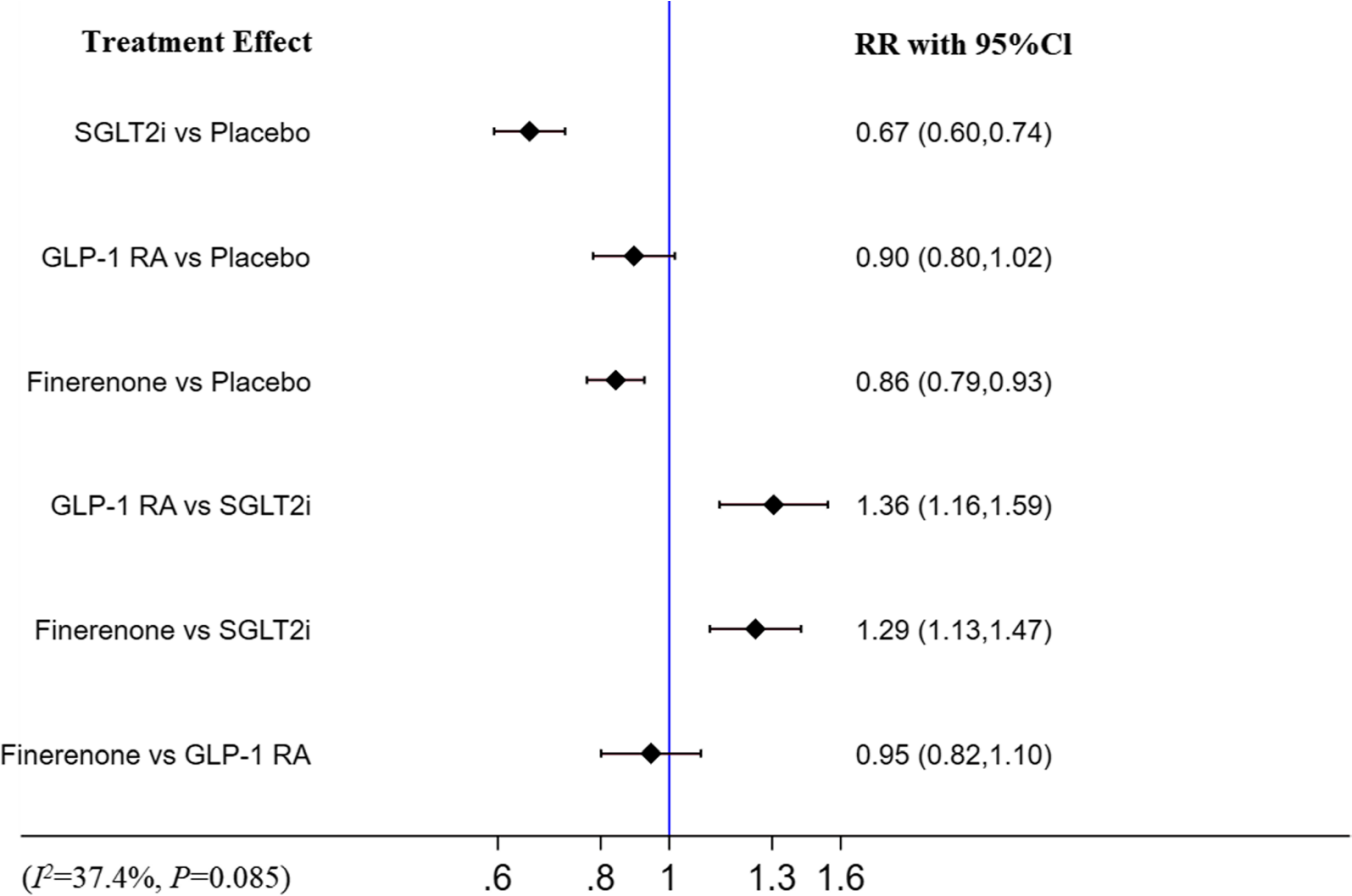
Network meta-analysis reporting RR for renal outcome in patients with T2DM and CKD

Finerenone (RR [95% CI]; 0.86 [0.79–0.93]) and SGLT-2i (RR [95% CI]; 0.67 [0.60–0.74]) significantly decreased the morbidity of renal outcome when compared with placebo, while GLP-1 RA (RR [95% CI]; 0.90 [0.73–1.02]) did not. Compared with finerenone (finerenone vs SGLT2i: RR [95% CI]; 1.31 [1.07–1.61]) and GLP-1 RA (GLP-1 RA vs SGLT2i: RR [95% CI]; 1.49 [1.18–1.89]), SGLT-2i were associated with a decreased morbidity of renal outcome. Finerenone was comparable to GLP-1 RA (RR [95% CI]; 0.95 [0.82–1.10]). There was moderate heterogeneity (I^2^ = 37.4%, p = 0.085). The detail is shown in Fig. 5.

### HHF

**Fig.6:**
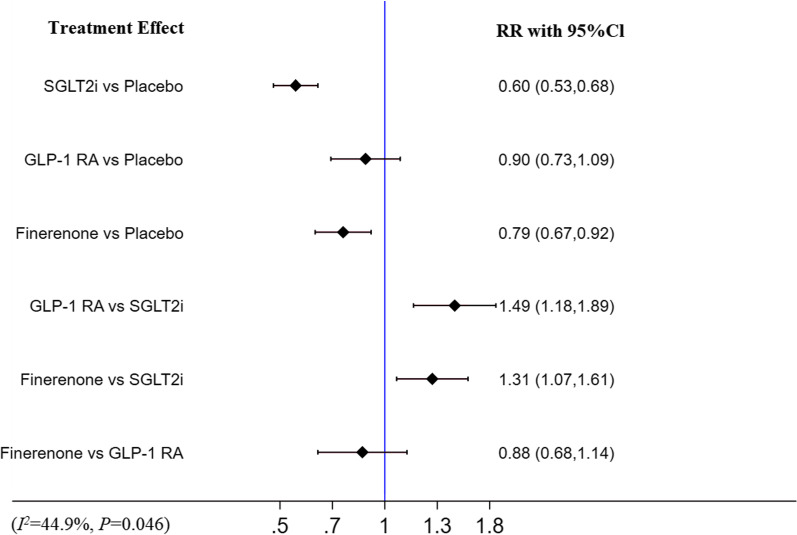
Network meta-analysis reporting RR for HHF in patients with T2DM and CKD

Compared with placebo, finerenone (RR [95% CI]; 0.79 [0.67–0.92]) and SGLT2i (RR [95% CI]; 0.60 [0.53–0.68]) were associated with a decreased risk of HHF while GLP-1 RA (RR [95% CI]; 0.90 [0.73–1.09]) did not. Compared with finerenone (finerenone vs SGLT2i: RR [95% CI]; 1.31 [1.07–1.61]) and GLP-1 RA (GLP-1 RA vs SGLT2i: RR [95% CI]; 1.49 [1.18–1.89]), SGLT-2i was shown to be significantly more effective in reducing HHF. But there was no significant difference in the risk of HHF between finerenone and GLP-1 RA (RR [95% CI]; 0.88 [0.68–1.14]). There was moderate heterogeneity (I^2^ = 44.9%, p = 0.046). The detail is shown in Fig. 6.

### ACD

**Fig.7:**
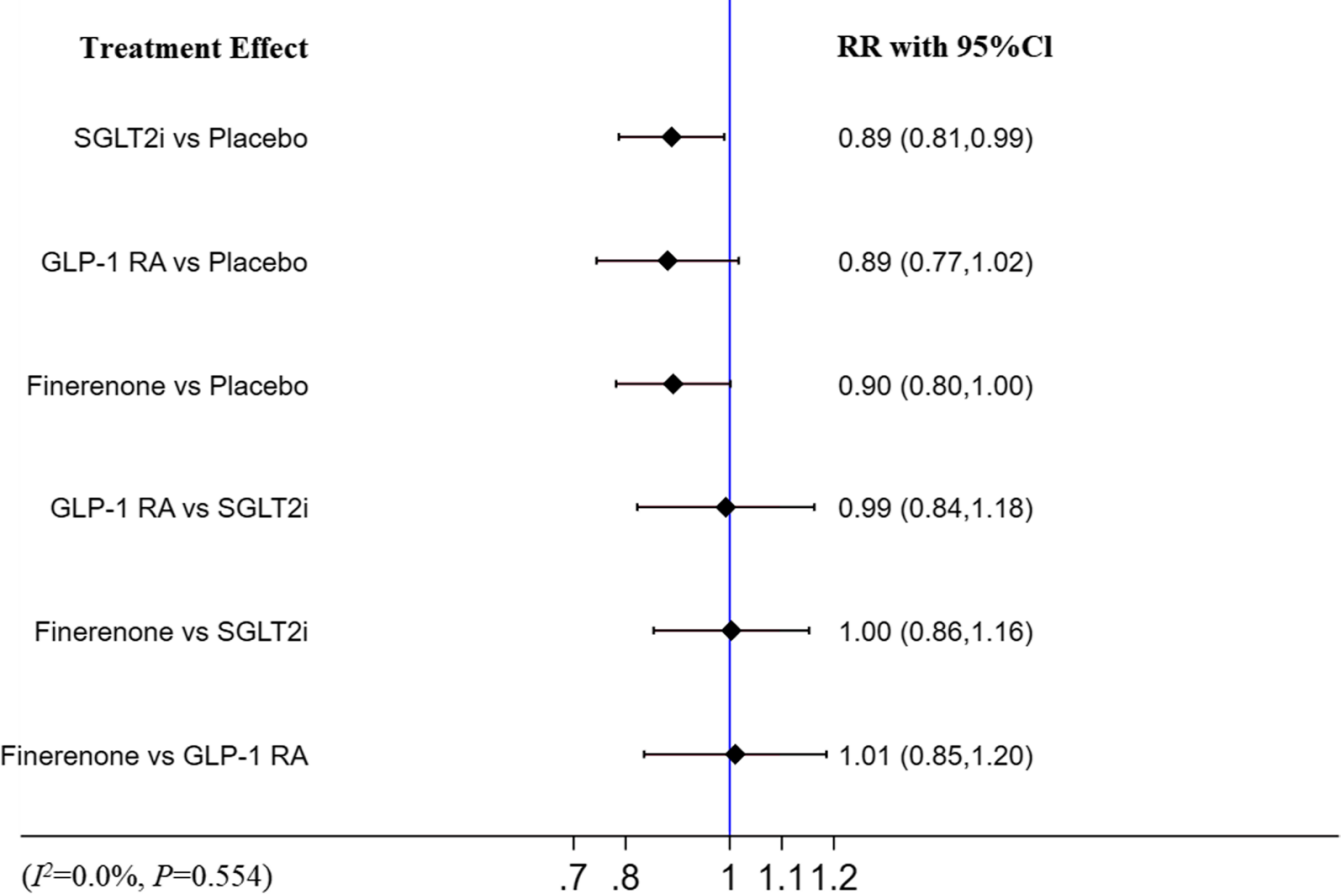
Network meta-analysis reporting RR for ACD in patients with T2DM and CKD

Compared with placebo, finerenone (RR [95% CI]; 0.90 [0.80–1.00) had a tendency to decrease the risk of ACD and SGLT-2i (RR [95% CI]; 0.89 [0.81–0.99]) were associated with a decreased risk of ACD, while GLP-1 RA (RR [95% CI]; 0.89 [0.77–1.02]) did not. There was no significant difference among finerenone, SGLT2i and GLP-1RA (RR 0.99, 95% CI 0.84–1.18; RR 1.00, 95% CI 0.86–1.16; RR 1.01, 95% CI 0.85–1.20, respectively). This analysis showed no heterogeneity (I^2^ = 0.0%, p = 0.554). The detail is shown in Fig. 7.

### CVD

**Fig.8:**
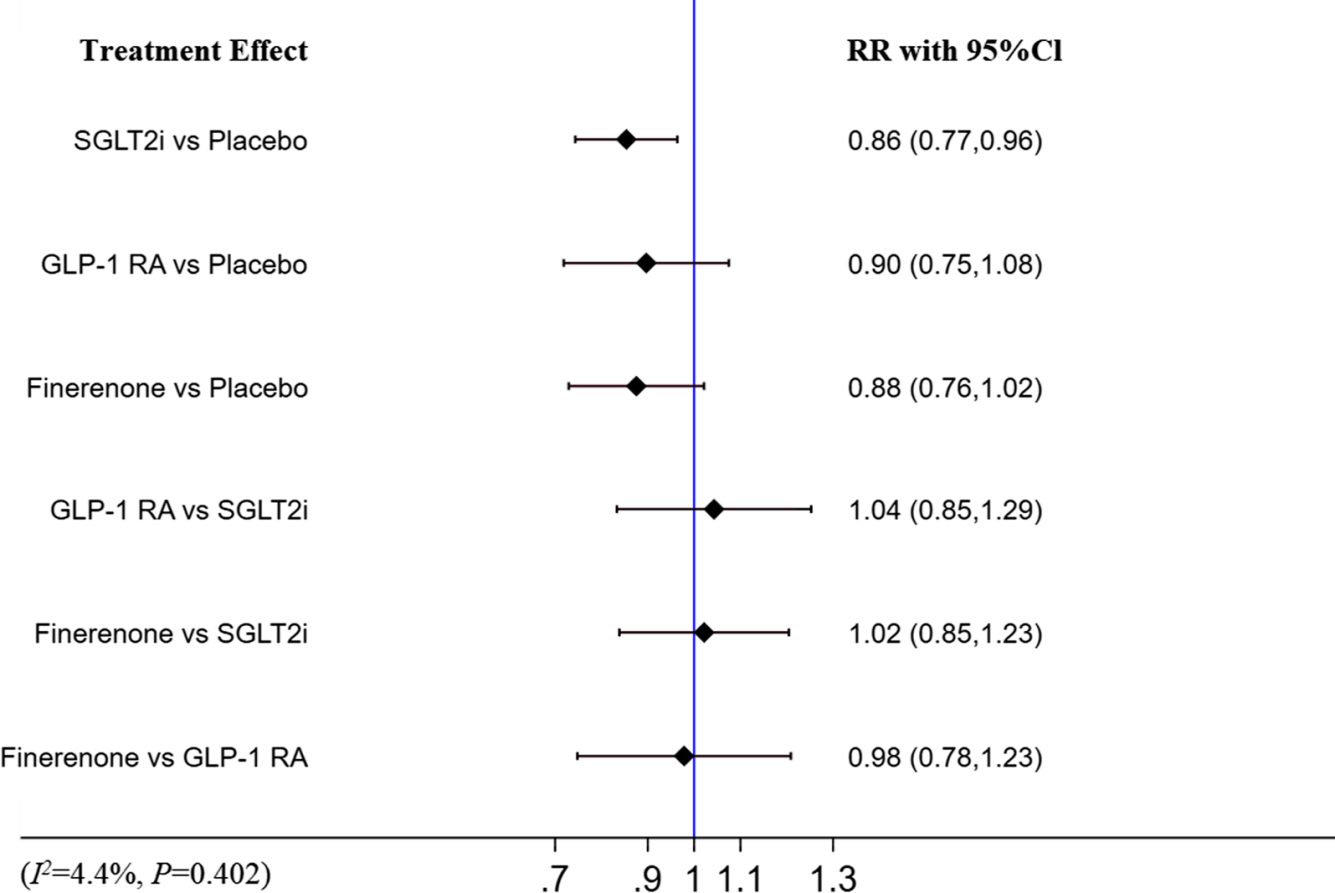
Network meta-analysis reporting RR for CVD in patients with T2DM and CKD

As for CVD, only SGLT-2i were associated with a decreased events (RR [95% CI]; 0.86, [0.77–0.96]) compared with placebo. There was no significant difference between finerenone and placebo, GLP-1 RA and placebo. And finerenone, SGLT2i and GLP-1 RA were comparable in reducing the risk of CVD. (Fig. 8). The analysis of CVD showed no heterogeneity (I^2^ = 4.4%, p = 0.402). The detail is shown in Fig. 8.

### Finerenone vs 10 interventions included in SGLT2i and GLP-1 RA

In order to provide more specific recommendations for clinical drug selection, we further evaluated the efficacy of finerenone and the 10 interventions included in SGLT2i and GLP-1 RA. As for MACE, finerenone was comparable to other interventions, except liraglutide (RR [95% CI]; 1.28 [1.04–1.56]). Canagliflozin, sotagliflozin, efpeglenatide and liraglutide were associated with a decreased risk of MACE compared to ertugliflozin or exenatide. Liraglutide had a tendency to reduce MACE compared to albiglutide (RR [95% CI]; 0.74 [0.55–1.00]), it also showed more positive influence when compared with dapagliflozin (RR [95% CI]; 0.75 [0.58–0.96]). Compared to placebo, finerenone (RR [95% CI]; 0.88 [0.80–0.97]), canagliflozin(RR [95% CI]; 0.78 [0.68–0.89]), sotagliflozin(RR [95% CI]; 0.76 [0.66–0.87]) efpeglenatide(RR [95% CI]; 0.70 [0.53–0.90]) and liraglutide(RR [95% CI]; 0.69 [0.58–0.82]) displayed significant effect when reducing of MACE, while other interventions were not. The detail is shown in Table 5.

**Table 5:** Pairwise league table of MACE and HHF

Comparisons for MACE (bottom left) of the 11 interventions and HHF (upper right) of the 8 interventions. RR with 95% CI											
EM	1.01 (0.65,1.59)	0.94 (0.57,1.55)	0.85 (0.49,1.50)	1.12 (0.71,1.78)	1.33 (0.87,2.03)		1.85 (1.14,3.01)		1.24 (0.76,2.00)		1.68 (1.14,2.50)
1.14 (0.88,1.49)	CA	0.93 (0.64,1.35)	0.84 (0.53,1.33)	1.11 (0.80,1.53)	1.31 (1.00,1.71)		1.83 (1.28,2.61)		1.22 (0.86,1.74)		1.66 (1.34,2.06)
0.96 (0.72,1.29)	0.84 (0.67,1.06)	DA	0.91 (0.55,1.51)	1.19 (0.81,1.76)	1.41 (1.00,2.00)		1.97 (1.30,2.99)		1.31 (0.87,1.99)		1.79 (1.32,2.44)
0.81 (0.59,1.13)	0.71 (0.54,0.93)	0.84 (0.63,1.13)	ER	1.32 (0.82,2.10)	1.55 (1.01,2.39)		2.17 (1.33,3.54)		1.45 (0.89,2.36)		1.97 (1.32,2.95)
1.17 (0.90,1.53)	1.02 (0.85,1.24)	1.22 (0.97,1.52)	1.44 (1.10,1.89)	SO	1.18 (0.89,1.58)		1.65 (1.14,2.39)		1.10 (0.76,1.59)		1.50 (1.18,1.91)
1.01 (0.79,1.29)	0.88 (0.75,1.04)	1.05 (0.85,1.29)	1.24 (0.96,1.60)	0.86 (0.73,1.02)	FI		1.40 (1.01,1.93)		0.93 (0.68,1.29)		1.27 (1.08,1.49)
0.96 (0.69,1.33)	0.84 (0.64,1.10)	1.00 (0.74,1.34)	1.18 (0.85,1.65)	0.82 (0.62,1.07)	0.95 (0.73,1.23)	AL					
0.86 (0.66,1.13)	0.75 (0.62,0.92)	0.89 (0.71,1.13)	1.06 (0.80,1.40)	0.74 (0.60,0.90)	0.85 (0.71,1.02)	0.90 (0.68,1.19)	EX		0.67 (0.45,0.99)		0.91 (0.69,1.21)
1.08 (0.73,1.58)	0.94 (0.67,1.32)	1.12 (0.78,1.60)	1.32 (0.89,1.96)	0.92 (0.65,1.29)	1.06 (0.77,1.48)	1.12 (0.76,1.66)	1.25 (0.88,1.77)	SE			
1.29 (0.97,1.72)	1.13 (0.90,1.41)	1.34 (1.04,1.73)	1.59 (1.18,2.13)	1.10 (0.88,1.38)	1.28 (1.04,1.56)	1.35 (1.00,1.81)	1.50 (1.19,1.89)	1.20 (0.84,1.72)	LI		1.14 (0.93,1.41)
1.27 (0.88,1.82)	1.11 (0.81,1.51)	1.31 (0.94,1.84)	1.56 (1.08,2.25)	1.08 (0.79,1.48)	1.25 (0.93,1.69)	1.32 (0.91,1.91)	1.47 (1.07,2.02)	1.18 (0.77,1.80)	0.98 (0.70,1.37)	EF	
0.89 (0.71,1.12)	0.78 (0.68,0.89)	0.92 (0.77,1.11)	1.09 (0.87,1.38)	0.76 (0.66,0.87)	0.88 (0.80,0.97)	0.93 (0.73,1.18)	1.03 (0.89,1.20)	0.83 (0.60,1.13)	0.69 (0.58,0.82)	0.70 (0.53,0.93)	PL

RR with 95% CI

EM, empagliflozin; CA, canagliflozin; DA, dapagliflozin; ER, ertugliflozin; SO, sotagliflozin; FI, finerenone; AL, albiglutide; EX, exenatide; SE, semaglutide; LI, liraglutide; EF, efpeglenatide; PL, placebo

In renal outcome, the results of comparison showed that empagliflozin (RR [95% CI]; 0.76 [0.63–0.93]), canagliflozin (RR [95% CI]; 0.81 [0.67–0.99]) and dapagliflozin (RR [95% CI]; 0.70 [0.55–0.87]) significantly reduced the morbidity of renal outcome compared to finerenone. Finerenone, empagliflozin, canagliflozin and dapagliflozin reduced renal events significantly compared to placebo. The detail is shown in Table 6.

**Table 6:** Pairwise league table of renal outcome

Comparisons for renal outcome of the 8 interventions								
Empagliflozin	1.07 (0.83,1.37)	0.91 (0.69,1.20)	1.26 (0.84,1.87)	1.31 (1.08,1.59)	1.38 (1.09,1.75)	1.50 (1.02,2.19)	1.34 (1.02,1.76)	1.53 (1.28,1.83)
0.94 (0.73,1.20)	Canagliflozin	0.85 (0.65,1.13)	1.18 (0.79,1.76)	1.23 (1.01,1.50)	1.30 (1.02,1.65)	1.40 (0.96,2.06)	1.25 (0.95,1.65)	1.44 (1.20,1.72)
1.10 (0.83,1.45)	1.17 (0.89,1.55)	Dapagliflozin	1.38 (0.91,2.09)	1.44 (1.14,1.81)	1.52 (1.16,1.98)	1.64 (1.10,2.45)	1.47 (1.09,1.98)	1.68 (1.36,2.08)
0.80 (0.53,1.19)	0.85 (0.57,1.27)	0.72 (0.48,1.10)	Sotagliflozin	1.04 (0.72,1.51)	1.10 (0.74,1.63)	1.19 (0.73,1.95)	1.06 (0.70,1.61)	1.22 (0.85,1.74)
0.76 (0.63,0.93)	0.81 (0.67,0.99)	0.70 (0.55,0.87)	0.96 (0.66,1.39)	Finerenone	1.06 (0.88,1.26)	1.14 (0.81,1.62)	1.02 (0.82,1.28)	1.17 (1.08,1.27)
0.72 (0.57,0.92)	0.77 (0.61,0.98)	0.66 (0.51,0.86)	0.91 (0.61,1.35)	0.95 (0.79,1.14)	Dulaglutide	1.08 (0.75,1.57)	0.97 (0.74,1.26)	1.11 (0.94,1.30)
0.67 (0.46,0.98)	0.71 (0.49,1.04)	0.61 (0.41,0.91)	0.84 (0.51,1.37)	0.87 (0.62,1.24)	0.92 (0.64,1.34)	Exenatide	0.89 (0.60,1.33)	1.02 (0.73,1.43)
0.75 (0.57,0.98)	0.80 (0.61,1.05)	0.68 (0.51,0.92)	0.94 (0.62,1.42)	0.98 (0.78,1.23)	1.03 (0.79,1.34)	1.12 (0.75,1.66)	Liraglutide	1.14 (0.93,1.41)
0.65 (0.55,0.78)	0.70 (0.58,0.83)	0.59 (0.48,0.74)	0.82 (0.57,1.18)	0.86 (0.79,0.93)	0.90 (0.77,1.06)	0.98 (0.70,1.37)	0.87 (0.71,1.08)	Placebo

RR with 95% CI

Finerenone (RR [95% CI]; 0.72 [0.52–0.99]), empagliflozin (RR [95% CI]; 0.54 [0.33–0.88]), canagliflozin (RR [95% CI]; 0.55 [0.38–0.78]), dapagliflozin (RR [95% CI]; 0.51 [0.33–0.77]), ertugliflozin (RR [95% CI]; 0.46 [0.28–0.75]), sotagliflozin (RR [95% CI]; 0.61 [0.42–0.88]) and liraglutide (RR [95% CI]; 0.67 [0.45–0.99]) significantly reduced HHF compared to exenatide. At the same time, all 7 interventions mentioned above significantly reduced HHF compared to placebo (Table 5). Another discovery worth noting is that canagliflozin (RR [95% CI]; 0.76 [0.58–1.00]) and dapagliflozin (RR [95% CI]; 0.71 [0.50–1.00]) had a tendency to decrease HHF compared to finerenone, and finerenone was associated with a higher risk of HHF than ertugliflozin (RR [95% CI]; 1.55 [1.01–2.39]). The detail is shown in Table 5.

When it comes to ACD, finerenone was comparable to other interventions. And finerenone (RR [95% CI]; 0.90 [0.80–1.00]) tended to reduce the risk of ACD when compared with placebo, while dapagliflozin (RR [95% CI]; 0.81 [0.66–0.98]) and liraglutide (RR [95% CI]; 0.76 [0.62–0.93]) had significant effect than placebo. As for CVD, liraglutide (RR [95% CI]; 0.69 [0.52–0.90]) was better than placebo, while other interventions were not. And finerenone was also comparable to other interventions. The detail is shown in Table 7.

**Table 7:** Pairwise league table of ACD and CVD

Comparisons for ACD (bottom left) and CVD (upper right) of the 8 interventions								
Empagliflozin	1.11 (0.74,1.65)	1.09 (0.70,1.70)	1.12 (0.71,1.77)	1.14 (0.76,1.72)	1.13 (0.77,1.65)	1.40 (0.92,2.14)	0.88 (0.56,1.37)	1.28 (0.90,1.81)
1.02 (0.67,1.56)	Canagliflozin	0.99 (0.71,1.38)	1.02 (0.72,1.44)	1.03 (0.77,1.37)	1.02 (0.80,1.30)	1.27 (0.94,1.72)	0.79 (0.57,1.11)	1.16 (0.95,1.40)
1.06 (0.69,1.62)	1.04 (0.78,1.37)	Dapagliflozin	1.03 (0.69,1.54)	1.04 (0.74,1.48)	1.03 (0.76,1.41)	1.28 (0.89,1.84)	0.80 (0.54,1.18)	1.17 (0.89,1.54)
0.73 (0.41,1.30)	0.72 (0.45,1.16)	0.70 (0.43,1.12)	Ertugliflozin	1.01 (0.71,1.46)	1.00 (0.72,1.39)	1.25 (0.86,1.81)	0.78 (0.52,1.16)	1.14 (0.85,1.52)
0.86 (0.57,1.31)	0.85 (0.65,1.10)	0.82 (0.63,1.06)	1.18 (0.74,1.87)	Sotagliflozin	0.99 (0.76,1.28)	1.23 (0.90,1.69)	0.77 (0.54,1.09)	1.12 (0.91,1.39)
0.95 (0.64,1.41)	0.93 (0.74,1.16)	0.90 (0.72,1.13)	1.29 (0.83,2.02)	1.10 (0.90,1.35)	Finerenone	1.24 (0.94,1.64)	0.78 (0.57,1.06)	1.13 (0.98,1.31)
0.84 (0.55,1.28)	0.82 (0.63,1.08)	0.79 (0.61,1.04)	1.14 (0.71,1.83)	0.97 (0.76,1.25)	0.88 (0.71,1.10)	Exenatide	0.62 (0.43,0.90)	0.91 (0.72,1.15)
1.13 (0.73,1.73)	1.11 (0.83,1.47)	1.07 (0.80,1.42)	1.53 (0.95,2.48)	1.31 (1.00,1.70)	1.19 (0.94,1.50)	1.34 (1.02,1.77)	Liraglutide	1.46 (1.11,1.92)
0.85 (0.58,1.24)	0.83 (0.69,1.02)	0.81 (0.66,0.98)	1.16 (0.75,1.79)	0.99 (0.83,1.17)	0.90 (0.80,1.00)	1.01 (0.84,1.22)	0.76 (0.62,0.93)	Placebo

RR with 95% CI

### Conclusions from minimally contextualized framework

As for MACE and CVD, liraglutide could be considered as one of the most effective treatment currently available. Efpeglenatide, sotagliflozin, canagliflozin and finerenone could be considered as inferior to the most effective in reducing the risk of MACE. In renal outcome, dapagliflozin, empagliflozin and canagliflozin could be considered as the most effective, while finerenone could be considered as inferior to the most effective. When it comes to HHF, ertugliflozin could be considered as the most effective. Liraglutide and dapagliflozin could be considered as the most effective in reducing the incidence of ACD. As was presented in Table 8.

**Table 8:** Final classification of 11 interventions, based on NMA of interventions for patients with T2DM and CKD

Certainty of the evidence	Category	Intervention	Intervention vs placebo RR (95% CI)	Surface under the cumulative ranking curve
*MACE*				
High certainty (moderate to high certainty evidence)	Category 2: among the most effective	Liraglutide	0.69 (0.58,0.82)	0.904
	Category 1: inferior to the most effective, or superior to the least effective	Efpeglenatide	0.70 (0.53,0.93)	0.850
		Sotagliflozin	0.76 (0.66,0.87)	0.773
		Canagliflozin	0.78 (0.68,0.89)	0.732
		Finerenone	0.88 (0.80,0.97)	0.484
	Category 0: among the least effective	Semaglutide	0.83 (0.60,1.13)	0.589
		Empagliflozin	0.58 (0.25,1.36)	0.456
		Dapagliflozin	0.92 (0.77,1.11)	0.381
		Albiglutide	0.93 (0.73,1.18)	0.374
		Exenatide	1.03 (0.89,1.20)	0.151
		Ertugliflozin	1.09 (0.87,1.38)	0.107
Low certainty (low to very low certainty evidence)	Category 0/1: might be among the most/least effective	–	–	–
*Renal outcome*				
High certainty (moderate to high certainty evidence)	Category 2: among the most effective	Dapagliflozin	0.59 (0.48,0.74)	0.941
		Empagliflozin	0.65 (0.55,0.78)	0.847
		Canagliflozin	0.70 (0.58,0.83)	0.765
	Category 1: inferior to the most effective, or superior to the least effective	Finerenone	0.86 (0.79,0.93)	0.437
	Category 0: among the least effective	Sotagliflozin	0.82 (0.57,1.18)	0.489
		Liraglutide	0.87 (0.71,1.08)	0.386
		Dulaglutide	0.90 (0.77,1.16)	0.321
		Exenatide	0.98 (0.70,1.37)	0.216
Low certainty (low to very low certainty evidence)	Category 0/1: might be among the most/least effective	–	–	–
*HHF*				
High certainty (moderate to high certainty evidence)	Category 2: among the most effective	Ertugliflozin	0.51 (0.34,0.76)	0.863
	Category 1: inferior to the most effective, or superior to the least effective	Empagliflozin	0.59 (0.40,0.88)	0.702
		Dapagliflozin	0.56 (0.41,0.76)	0.785
		Canagliflozin	0.60 (0.48,0.75)	0.703
		Sotagliflozin	0.67 (0.52,0.85)	0.557
		Liraglutide	0.73 (0.56,0.97)	0.427
		Finerenone	0.79 (0.67,0.92)	0.327
	Category 0: among the least effective	Exenatide	1.10 (0.83,1.46)	0.040
Low certainty (low to very low certainty evidence)	Category 0/1: might be among the most/least effective	–	–	–
*ACD*				
High certainty (moderate to high certainty evidence)	Category 1: among the most effective	Liraglutide	0.76 (0.62,0.93)	0.872
		Dapagliflozin	0.81 (0.66,0.98)	0.769
	Category 0: among the least effective	Canagliflozin	0.83 (0.69,1.02)	0.700
		Empagliflozin	0.85 (0.58,1.42)	0.624
		Finerenone	0.90 (0.80,1.00)	0.553
		Sotagliflozin	0.99 (0.83,1.17)	0.315
		Exenatide	1.01 (0.84,1.22)	0.258
		Ertugliflozin	1.16 (0.75,1.79)	0.156
Low certainty (low to very low certainty evidence)	Category 0/1: might be among the most/least effective	–	–	–
*CVD*				
High certainty (moderate to high certainty evidence)	Category 1: among the most effective	Liraglutide	0.69 (0.52,0.90)	0.907
	Category 0: among the least effective	Empagliflozin	0.78 (0.55,1.11)	0.704
		Dapagliflozin	0.85 (0.65,1.13)	0.565
		Canagliflozin	0.87 (0.71,1.05)	0.554
		Finerenone	0.88 (0.76,1.02)	0.510
		Ertugliflozin	0.88 (0.66,1.18)	0.507
		Sotagliflozin	0.89 (0.72,1.10)	0.484
		Exenatide	1.10 (0.87,1.39)	0.091
Low certainty (low to very low certainty evidence)	Category 0/1: might be among the most/least effective	–	–	–

### Sensitivity analyses

The results of sensitivity analyses are summarized in Table 9. We conducted a sensitivity analysis excluding “Cherney 2021”, as Cherney 2021 only included diabetics with severe CKD (eGFR: 15–30 ml/min/1.73 m^2^). In MACE, renal outcome and ACD, the results of sensitivity analyses were comparable to non-exclusion of “Cherney 2021”. Compared to sotagliflozin, liraglutide (RR [95% CI]; 0.76 [0.58–0.99]) was associated with a decreased risk of ACD. Whereas the previous results showed liraglutide had a trend towards a reduction in CVD compared to sotagliflozin.

**Table 9:** The summary of sensitivity analyses

Outcomes	Finerenone		SGLT-2i		GLP-1 RA		Comparison	Risk ratio	95%Cl	I^2^(%)	P
	I	C	I	C	I	C					
MACE	6519	6507	12,959	11,595	6045	5344	SGLT-2i vs placebo	0.84	0.78–0.91	23.0	0.199
							GLP-1 RA vs placebo	0.86	0.78–0.94		
							Finerenone vs placebo	0.88	0.80–0.97		
							GLP-1 RA vs SGLT-2i	1.02	0.90–1.15		
							Finerenone vs SGLT-2i	1.05	0.93–1.18		
							Finerenone vs GLP-1 RA	1.03	0.90–1.17		
Renal outcome	7234	6600	11,547	10,888	3754	3780	SGLT-2i vs placebo	0.66	0.59–0.73	39.2	0.079
							GLP-1 RA vs placebo	0.90	0.80–1.02		
							Finerenone vs placebo	0.86	0.79–0.93		
							GLP-1 RA vs SGLT-2i	1.37	1.17–1.61		
							Finerenone vs SGLT-2i	1.30	1.14–1.49		
							Finerenone vs GLP-1 RA	0.95	0.82–1.10		
HHF	6519	6507	12,960	11,596	2673	2662	SGLT-2i vs placebo	0.60	0.53–0.68	49.9	0.030
							GLP-1 RA vs placebo	0.90	0.73–1.09		
							Finerenone vs placebo	0.79	0.67–0.92		
							GLP-1 RA vs SGLT-2i	1.49	1.18–1.89		
							Finerenone vs SGLT-2i	1.31	1.07–1.61		
							Finerenone vs GLP-1 RA	0.88	0.68–1.14		
ACD	6519	6507	11,944	10,805	2673	2662	SGLT-2i vs placebo	0.90	0.81–0.99	0.0	0.537
							GLP-1 RA vs placebo	0.89	0.77–1.02		
							Finerenone vs placebo	0.90	0.80–1.00		
							GLP-1 RA vs SGLT-2i	0.99	0.83–1.17		
							Finerenone vs SGLT-2i	1.00	0.86–1.16		
							Finerenone vs GLP-1 RA	1.01	0.85–1.20		
CVD	6519	6507	12,960	11,596	2673	2662	SGLT-2i vs placebo	0.87	0.78–0.97	0.0	0.658
							GLP-1 RA vs placebo	0.90	0.75–1.08		
							Finerenone vs placebo	0.88	0.76–1.02		
							GLP-1 RA vs SGLT-2i	1.04	0.84–1.28		
							Finerenone vs SGLT-2i	1.02	0.85–1.22		
							Finerenone vs GLP-1 RA	0.98	0.78–1.23		

*I* intervention, *C* control

## Discussion

In the absence of RCT directly comparing to nonsteroidal and selective mineralocorticoid receptor antagonists, SGLT2i and GLP-1 RA, this network meta-analysis evaluated the relative efficacy of three drugs on cardiovascular and renal outcomes in patients with T2DM and CKD. This network meta-analysis was based on 18 large trials, which included 51,496 patients randomly assigned to finerenone, SGLT2i, GLP-1 RA or placebo. Our results revealed that finerenone can decrease the risk of MACE, renal outcome and HHF, alongside with the tendency to reduce ACD in patients with T2DM and CKD. Our study found that finerenone has the advantage reducing MACE risk just as well as SGLT2i, which was inconsistent with another network meta-analysis [65]. The cause may be that that research only included one trial correlating to finerenone (FIDELIO-DKD) and had the possibility of small-sample bias. SGLT2i was found to be comprehensive in reducing the risk of MACE, renal outcome, HHF, CVD and ACD. It outperformed finerenone in terms of reducing the risk of renal outcome.

This study revealed that GLP-1 RA decreased the risk of MACE compared with placebo, which varied with another network meta-analysis [66]. The inconsistency may be due to the exclusion of ELIXA trial in that article, as its definition of MACE included unstable angina(so why leading to the unsignificant/significant result?). In addition, GLP-1 RA did not show any significant benefit in reducing renal outcome when compared with placebo. Our study also revealed that SGLT-2i were associated with a decreased risk of renal outcome and HHF compared with finerenone and GLP-1 RA. This seemed to imply that GLP1-RA has no significant advantage over SGLT2i, but analysis between finerenone and 10 interventions included in SGLT2i and GLP-1 RA showed different results. Liraglutide, one of GLP-1 RA, was associated with a decreased risk of MACE, ACD, CVD and HHF. Amongst all 11 interventions included in this study, liraglutide was the only intervention to show efficacy in CVD compared with placebo. As shown in minimally contextualized framework, liraglutide also ranked first in MACE, ACD and CVD among all 11 interventions included in this study. This could mean that liraglutide was a more preferable choice for DM patients with CKD who have an elevated risk of cardiovascular events.

Several mechanisms have been proposed for the positive impact of finerenone. As a nonsteroidal, selective mineralocorticoid receptor antagonist, finerenone has been shown to have potent anti-inflammatory and antifibrotic effects while reducing the urinary albumin-to-creatinine ratio, which may be related to its benefits in renal outcome and HHF [67–70].

As for the morbidity, renal outcome and HHF, it was clear that SGLT-2i had more significant impact than finerenone, which might be explained by the special potency of SGLT-2i such as reducing blood glucose, reducing oxidative stress, losing weight, reducing uric acid, controlling blood pressure and improving renal ultrafiltration and hypoxia [65, 71–79].

Interestingly, in the three observed outcomes of ACD, HHF and CVD, GLP-1 RA did not show a significant advantage over placebo, but liraglutide, a GLP-1 RA did. In addition, liraglutide had a more outstandig effect than exenatide in the morbidity of MACE, ACD, HHF, and CVD. Based on chemical structure, GLP-1 RA could be divided into two groups: incretin-mimetics (exendin-4 analogs) and human GLP-1 analogues. Exenatide is a synthetic exendin‑4 analogue and liraglutide is an acylated analogue of GLP‑1.

The mechanism of renoprotective action of GLP-1 analogues is not entirely clear. It was believed that GLP-1 analogues are metabolized in target tissues via the common proteolytic pathway of large proteins. Their large molecular size or noncovalent attachment to albumin can prevent them from being eliminated by the kidneys. However, exendin-4 analogues are metabolized and eliminated by the kidneys. Moreover, exendin-4 analogues are resistant to inactivation of dipeptidyl peptidase-4, while GLP-1 analogues are partially metabolized to metabolites, which may be related to the better benefits in cardioprotective effects of liraglutide than exenatide [80, 81].

Major strengths of this network meta-analysis are of the follwowing: first and foremost, it was the first to investigate the effect of finerenone, SGLT-2 inhibitors and GLP-1 RA on cardiovascular and renal outcomes in patients with T2DM and CKD. Secondly, the number of included studies and sample size was large and the statistical efficiency was reliable, which provided evidence for individualized drug administration in clinical practice of patients with T2DM and CKD. Last but not least, in the Chronic Kidney Disease and Risk Management: Standards of Medical Care in Diabetes-2022 [19], ADA preferably recommended SGLT-2 inhibitors and finerenone over GLP-1 RA in vulnerable population who were at increased risk for cardiovascular events or CKD progression. They also emphasized that finerenone should only be recommended when the patient has CKD, that are at an increased risk for cardiovascular events, chronic kidney disease progression or are unable to use SGLT2i. They also suggest the use of GLP-1 RA or SGLT-2i for individuals with T2DM with or at high risk for ASCVD, and/or CKD in the Pharmacologic Approaches to Glycemic Treatment: Standards of Medical Care in Diabetes—2022 [15]. Our study supported their recommendations, with additional evidence that finerenone is comparable with SGLT2i in reducing the risk of MACE, meaning that if cardiovascular risks become prominent, then SGLT2i, finerenone and GLP-1 analogues are all suitable options. When the risk of renal events rises, the SGLT2i becomes the appropriate recommendation. The GLP-1 analogues could reduce the risk of MACE, HHF, CVD, especially ACD, suggesting that GLP-1 analogues can be an alternative option in patients with T2DM and CKD. GLP-1 RA may be suggested for cardiovascular risk reduction if such risk is a predominant problem, as they reduce risks of cardiovascular events appear to possibly slow CKD progression. While there is clear cardiovascular risk reduction associated with GLP-1 RA use in patients with T2DM and CKD, the proof of benefit on renal outcome will come with the results of the ongoing FLOW (A Research Study to See How Semaglutide Works Compared with Placebo in People With Type 2 Diabetes and Chronic Kidney Disease) trial with injectable semaglutide [82].

This study had several limitations. Firstly, we conducted this network meta-analysis on the basis of indirect comparisons. Therefore, our results require validation by head-to-head trials comparing finerenone with SGLT2i and GLP-1 RA. Secondly, partial studies included in this paper are subgroup analysis of RCTs, there is still a concern that patients with T2DM and CKD may not be completely randomized. Thirdly, there were more patients involved in SGLT2i than GLP-1 RA and finerenone. In addition, the baseline eGFR of patients in “Cherney 2021” was different from other studies. Although we did not observe high heterogeneity, these imbalances may limit the statistical capabilities of network meta-analysis. Finally, we did not pay attention to albuminuria, so we could not investigate the effects of finerenone, SGLT2i and GLP-1 RA for albuminuria in diabetics with CKD.

## Conclusion

In patients with T2DM and CKD, finerenone led to a risk reduction in MACE, renal outcome and HHF, SGLT2i were associated with a decreased risk of cardiovascular and renal events. Finerenone had a tendency to decrease the risk of ACD. GLP-1 RA were associated with a decreased risk of MACE. Finerenone was comparable to SGLT2i in reducing the risk of MACE, CVD and ACD. As for renal outcome and HHF, SGLT2i had significant effect over finerenone and GLP-1 RA. Among GLP-1 RA, GLP-1 analogues showed significantly reduced cardiovascular events compared with exendin-4 analogues. Cardiovascular risks are common within diabetic patients with CKD, when such risk jeopardize the wellbeing of the patient, SGLT2i, finerenone and GLP-1 analogues are all apposite recommendations, but when the risk of renal events heightens, then SGLT2i will be the sole recommendation available.

## Supplementary Information

**Additional file 1.** RoB-2 evaluation.

**Additional file 2.** Publication bias.

## Data Availability

All data generated or analyzed during this study are included in this published article.

## Abbreviations

SGLT2i: Sodium-glucose cotransporter-2 inhibitors
GLP-1 RA: Glucagonlike peptide-1 receptor agonists
T2DM: Type 2 diabetes mellitus
CKD: Chronic kidney disease
RCTs: Randomized control trials
RR: Risk ratio
CI: Confidence interval
HHF: Hospitalization for heart failure
MACE: Major adverse cardiovascular events
ACD: All-cause death
CVD: Cardiovascular death
DM: Diabetes mellitus
ASCVD: Atherosclerotic cardiovascular disease
ESRD: End-stage renal disease
MI: Myocardial infarction
ADA: American Diabetes Association
eGFR: Estimated glomerular filtration rate
PRISMA: Preferred reporting items for systematic reviews and meta-analyses
GRADE: Grading of recommendations assessment, development, and evaluation
CrCl: Creatinine clearance
UACR: Urinary albumin-to-creatinine ratio
